# Identifying potential RNAi targets in grain aphid (*Sitobion avenae* F.) based on transcriptome profiling of its alimentary canal after feeding on wheat plants

**DOI:** 10.1186/1471-2164-14-560

**Published:** 2013-08-16

**Authors:** Min Zhang, Yuwen Zhou, Hui Wang, Huw Dylan Jones, Qiang Gao, Dahai Wang, Youzhi Ma, Lanqin Xia

**Affiliations:** 1 Institute of Crop Sciences, Chinese Academy of Agricultural Sciences (CAAS), 12 Zhongguancun South Street, Beijing 100081, China; 2 Beijing Genomics Institute-Shenzhen (BGI-Shenzhen), Chinese Academy of Sciences, Shenzhen 518083, China; 3 Rothamsted Research, Harpenden, Hertfordshire AL5 2JQ, UK

**Keywords:** Grain aphid (*Sitobion avenae* F.), Alimentary canal, Transcriptome profile, Double strand RNA (dsRNA), Artificial feeding assay, RNA interference (RNAi), Aphid control

## Abstract

**Background:**

The grain aphid (*Sitobion avenae* F.) is a major agricultural pest which causes significant yield losses of wheat in China, Europe and North America annually. Transcriptome profiling of the grain aphid alimentary canal after feeding on wheat plants could provide comprehensive gene expression information involved in feeding, ingestion and digestion. Furthermore, selection of aphid-specific RNAi target genes would be essential for utilizing a plant-mediated RNAi strategy to control aphids via a non-toxic mode of action. However, due to the tiny size of the alimentary canal and lack of genomic information on grain aphid as a whole, selection of the RNAi targets is a challenging task that as far as we are aware, has never been documented previously.

**Results:**

In this study, we performed *de novo* transcriptome assembly and gene expression analyses of the alimentary canals of grain aphids before and after feeding on wheat plants using Illumina RNA sequencing. The transcriptome profiling generated 30,427 unigenes with an average length of 664 bp. Furthermore, comparison of the transcriptomes of alimentary canals of pre- and post feeding grain aphids indicated that 5490 unigenes were differentially expressed, among which, diverse genes and/or pathways were identified and annotated. Based on the RPKM values of these unigenes, 16 of them that were significantly up or down-regulated upon feeding were selected for dsRNA artificial feeding assay. Of these, 5 unigenes led to higher mortality and developmental stunting in an artificial feeding assay due to the down-regulation of the target gene expression. Finally, by adding fluorescently labelled dsRNA into the artificial diet, the spread of fluorescence signal in the whole body tissues of grain aphid was observed.

**Conclusions:**

Comparison of the transcriptome profiles of the alimentary canals of pre- and post-feeding grain aphids on wheat plants provided comprehensive gene expression information that could facilitate our understanding of the molecular mechanisms underlying feeding, ingestion and digestion. Furthermore, five novel and effective potential RNAi target genes were identified in grain aphid for the first time. This finding would provide a fundamental basis for aphid control in wheat through plant mediated RNAi strategy.

## Background

Aphids are major agricultural pests which cause significant yield losses of the crop plants each year by inflicting damage, both through the direct effects of feeding and by vectoring debilitating plant viruses [1,2]. Annual worldwide crop losses due to aphids are estimated at hundreds of millions of dollars [3-5]. The aphid species infesting wheat in China include grain aphid (*Sitobion avenae* F.), greenbug (*Schizaphis graminum* Rondani), *Rhopalosiphum padi* Linnaeus and *Metopolophium dirhodum* Walker. Of these, the grain aphid is the most dominant and destructive, affecting 65% of wheat production areas in China [6]. Grain aphid is also the major pest of wheat in Europe and North America [7]. For the last few decades, conventional breeding programs have been undertaken in an attempt to increase wheat aphid resistance worldwide [7]. However, due to the lack of effective aphid resistant germplasm, the complexity of plant-aphid interactions and the rapid development of resistant pest biotypes, outbreak of aphids causing substantial losses of wheat continue to be reported regularly [7,8]. Breeders and growers are still struggling to find an efficient genetic strategy for aphid control in wheat [8].

Expression in transgenic plants of double strand (dsRNA) designed against insect target genes has been shown to give protection against pests through RNA interference (RNAi), opening the way for a new generation of insect-resistant crops [9-11]. In the case of plant-mediated RNAi for insect control, both cell-autonomous and non-cell-autonomous RNAi are required for the persistence of RNAi effect. For cell-autonomous RNAi, the silencing process is limited to the cell in which the dsRNA is introduced, expressed and encompasses the RNAi process within individual cells [12-16]. The interfering effect of non-cell-autonomous RNAi, can take place in tissues/cells different from the location of application or production of the dsRNA. There are two different kinds of non-cell-autonomous RNAi: environmental RNAi and systemic RNAi. Environmental RNAi describes all processes in which dsRNA is taken up by a cell from the environment [17]. Systemic RNAi refers to all processes in which the silencing signal is transported from the cell in which the dsRNA is applied or expressed to other cells and tissues in which the silencing could take place [18-20]. In multicellular organisms, environmental RNAi can be followed by systemic RNAi and non-cell-autonomous RNAi will always be followed by cell-autonomous RNAi [12]. At least two mechanisms underlying RNAi in insects have been described so far, that is, the transmembrane channel-mediated uptake mechanism and an alternative endocytosis-mediated uptake mechanism [12]. In aphid, except the existence of SID-1 which is a multispan transmembrane protein mediating a systemic RNAi effect, the uptake mechanism of dsRNA remains to be determined [12,21]. Nevertheless, RNAi-mediated knockdown of *C002*, a gene strongly expressed in the salivary glands of *A. pisum* have led to death of aphids through direct injection of siRNA into aphid hemolymph [22]. V-ATPase is a membrane-bound protein that acts as a proton pump to establish the PH gradient within the gut lumen of many insects. Knockdown of *vATPase* transcripts following feeding on *vATPase* dsRNAs also led to significant mortality of *A. pisum*[23]. Furthermore, injection of dsRNA targeting genes encoding a calcium-binding protein calreticulin and a gut cathepsin, and feeding dsRNA of a water-specific aquaporin gene in artificial diet assay led to the down-regulation and malfunction of these targeted genes in *A. pisum*, although the target gene expression knockdown didn’t exceed 50% and was transient, persisting for less than a week [24,25]. So far, two cases of plant-mediated RNAi for aphid control have also been reported. Silencing *C002* gene and a gut-specific gene *Rack-1* in peach aphid resulted in the knock down of these two genes by up to 60% after feeding on transgenic tobacco and Arabidopsis plants, with affected aphids producing less progeny [26]. Host-generated siRNAs attenuated the expression of a serine proteinase gene in peach aphid, leading to a significant reduction in their fecundity and parthenogenetic population upon feeding on transgenic Arabidopsis plants [27]. These two cases exemplify the feasibility of plant-mediated RNAi approach for aphid control in agriculture. However, up to now, RNAi and plant-mediated RNAi for aphid control have not been documented in grain aphid nor has the systemic RNAi effect been observed previously in aphid species.

Illumina RNA sequencing (RNA-seq) enables the *de novo* reconstruction of the transcriptome for a non-model organism and also acts as a potentially effective way to identify RNAi targets by mass-screening of candidate genes in organisms with insufficient genomic information [28-32]. The midgut of Hemipterans and most other insects is lined by the perimicrovillar membrane (PMM) [33]. Hence, the midgut is the only portion of an insect’s body that has an active interface with the physical environment. The cells of the midgut, which are responsible for nutrient absorption from the gut lumen, can take up dsRNA and are one of the routes through which RNAi could be achieved in insects [12,34].

In this study, RNA-seq was used to investigate the transcriptome profile of the alimentary canal of grain aphid upon feeding on wheat plants. Comparison of the transcriptome profiles of the alimentary canals of pre- and post-feeding grain aphid on wheat plants revealed insights into the involvement of diversified genes and/or pathways. Furthermore, the systemic RNAi effect was investigated for the first time in grain aphid. Novel and effective RNAi target genes were explored through a dsRNA artificial diet feeding assay and candidate genes were identified that could act as potential targets for plant-mediated RNAi control of aphids in wheat.

## Results

### Analyses of the differentially expressed genes in the alimentary canal of grain aphid after feeding on wheat plants revealed the involvement of diversified genes and/or pathways in ingestion and digestion

To obtain a global view of the transcriptome profiles of the alimentary canals of grain aphids upon feeding, the alimentary canals of pre- and post-feeding grain aphids which were derived from a single clonal lineage were dissected for RNA isolation. High-throughput RNA-seq was performed by Illumina RNA sequencing in BGI-Shenzhen. After filtering out the adaptors and low quality sequences, 26.3 and 25.7 million clean reads with a total read-length over 2.4 and 2.3 billion nucleotides respectively, were obtained from the alimentary canals of grain aphids pre-feeding (DuiZhao) [NCBI: SRA accession number SRX216768] and 6 h after feeding (ChuLi) [NCBI: SRA accession number SRX216767]. These reads were subsequently assembled into contigs and then unigenes using SOAPdenovo software. In total, 32,714 and 35,267 unigenes were obtained for ChuLi and DuiZhao with a mean length of 501 and 493 bp, respectively. The unigenes from the two samples were then clustered using TGI clustering tools (TGICL) [35] and further assembled with Phrap Assembly Program (http://www.phrap.org), resulting in a total of 30,427 unigenes with mean length of 664 bp (Table 1). Of these unigenes, 20,736 (68.1%) had NCBI non-redundant protein database (nr) annotations as determined by BLAST search. Clusters of orthologous groups (COG), gene orthology (GO), and Kyoto Encyclopedia of Genes and Genomes (KEGG) annotations were performed to better understand the functions of these unigenes (data not shown).

**Table 1:** Summary of the transcriptomes of the alimentary canal of grain aphid upon feeding on wheat

	**Duizhao (pre-feeding)**	**Chuli (post-feeding)**	**All**
Total number of reads	26,285,658	25,667,932	
Total nucleotides (nt)	2,365,709,220	2,310,113,880	
Total number of contigs	93,174	83,219	
Total number of scaffolds	44,170	40,497	
Mean length of scaffolds (bp)	421	432	
Total unigenes	35,267	32,714	30,427
Mean length of unigenes (bp)	493	501	664
GC percentage	42.19%	44.12%	

**Figure 1:**
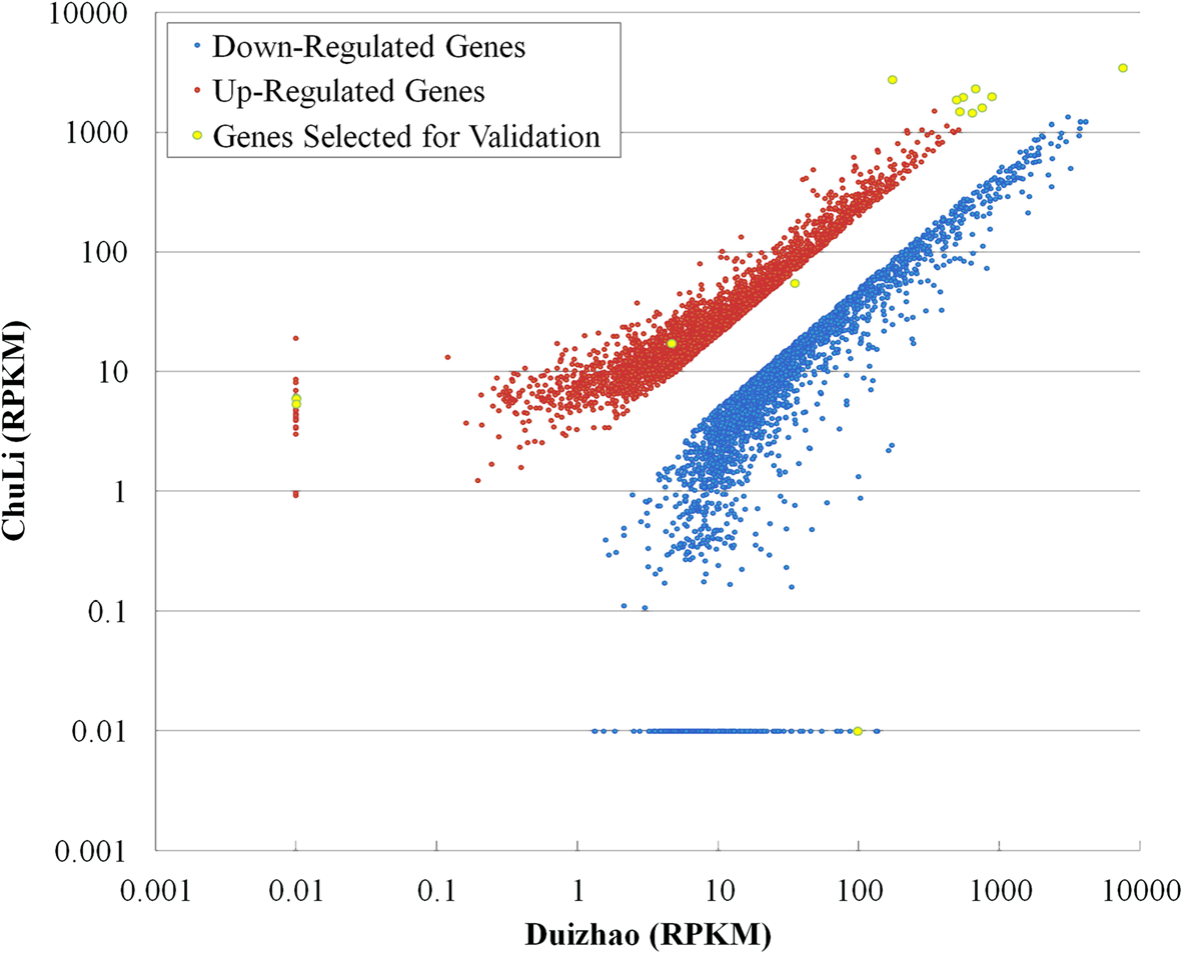
**The relative expression levels of differentially expressed genes in grain aphid alimentary canal upon feeding.** In total, 5490 genes were found to be differentially expressed among which up-regulated genes were indicated with purple points, whereas down-regulated genes with blue points. The selected 16 genes were indicated by yellow point.

The expression levels of the unigenes in post-feeding (Chuli) and pre-feeding (Duizhao) grain aphids were estimated based on RPKM (Reads Per kb per Million Reads) analysis [36]. In total, 5490 genes were found to be differentially expressed, among which, 2918 genes were up-regulated whereas 2572 genes were down-regulated (FDR < 0.001 and an absolute value of the log2 ratio > 1) (Figure 1). Besides, among the differentially expressed genes (DEGs), there were 34 genes only expressed in Chuli and 234 genes only expressed in Duizhao (See Additional file 1).

**Figure 2:**
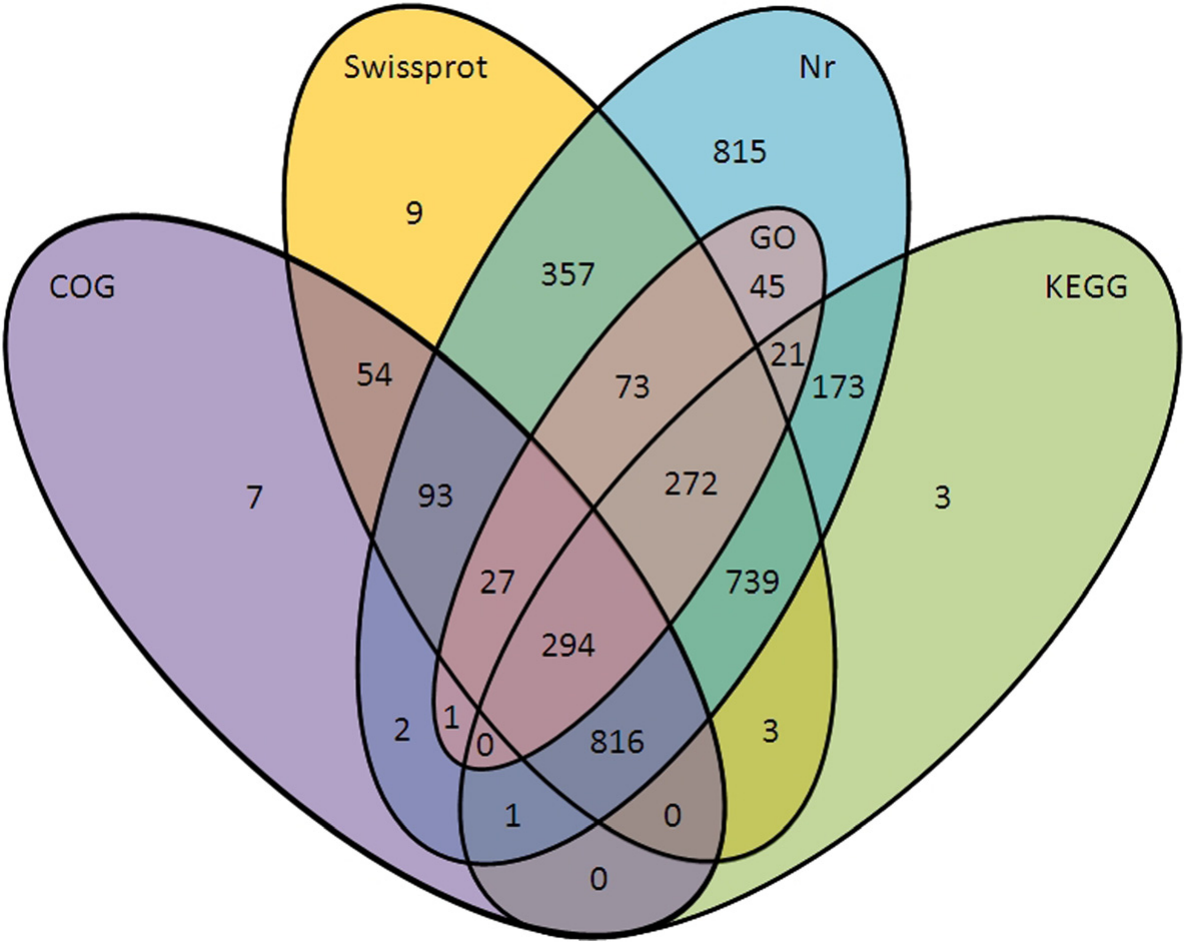
**A Venn diagram illustrating shared and unique DEGs annotated in nr, Swissprot, COG, GO and KEGG public databases.** Among 5490 DGEs, 3805 annotated in at least one of the public databases, including 3729, 237, 1295, 2322, 733 in nr, Swissprot, COG KEGG and GO databases, respectively.

For these DEGs, nr, COG, GO, and KEGG annotations were performed. Of these 5490 DEGs, only 3805 were annotated in at least one of the above public databases (Figure 2). Annotation details of these DEGs were as listed in Additional file 1. For example, only 733 had GO annotations (See Additional file 2). Whereas there were no presence of significantly enriched cellular components, three molecular functions were found to be enriched at p < 0.05 level, including NADH dehydrogenase activity, oxidoreductase activity, acting on NADH or NADPH, quinone or similar compound as acceptor, and NADH dehydrogenase (quinone) activity. Eight biological processes were significantly enriched (p < 0.05) including organ morphogenesis, organ development, generation of precursor metabolites and energy, imaginal disc development, cellular respiration, system development, oxidation-reduction process, energy derivation by oxidation of organic compounds. The enriched KEGG pathways upon feeding were as listed in Additional file 3. Among them, of the 2021 unigenes involved in ‘Metabolic pathways’ (ko01100), 420 showed differentially expressed manner, accounting for 18.09% of total DEGs (2322) with pathway annotation.

### Analyses of 16 highly up-regulated and down-regulated genes in grain aphid upon feeding and characterization of their tissue-specific expression manner

**Figure 3:**
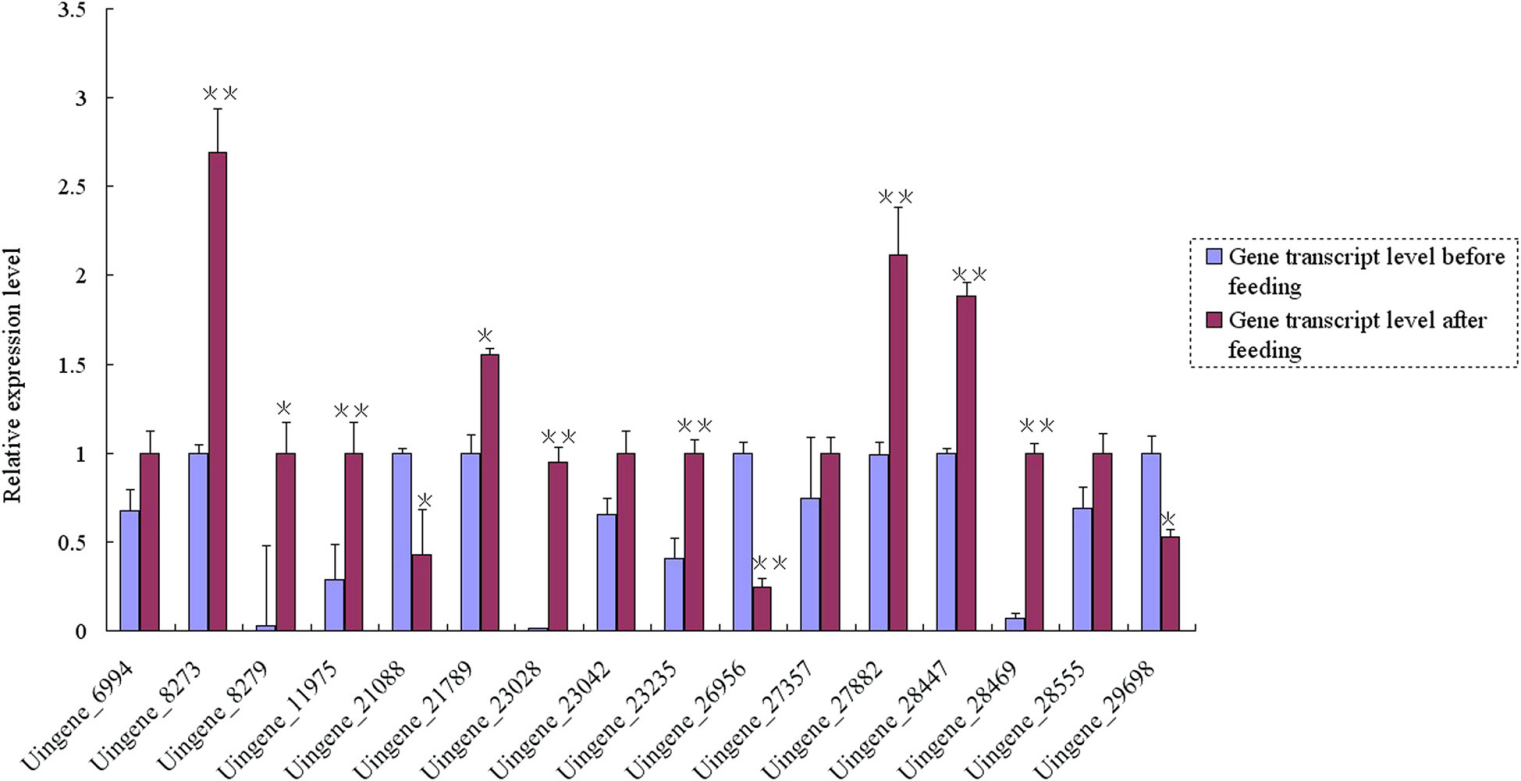
**qRT-PCR validation of the candidate RNAi target genes in grain aphid upon feeding.** (*Student’s t-test, n = 3, p < 0.05; **Student’s t-test, n = 3, p < 0.01).

**Figure 4:**
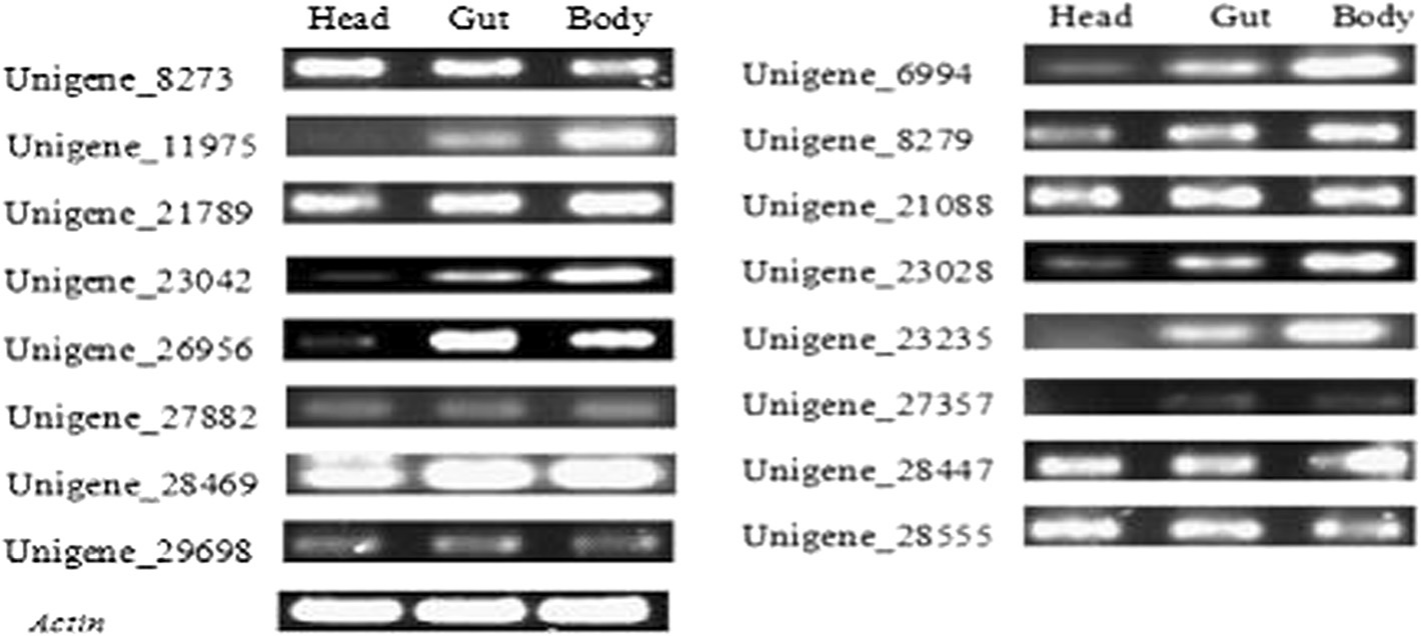
**Tissue-specific expression manner of 16 candidate RNAi target genes in grain aphid.** Semi-quantitative RT-PCRs were performed using the total RNA from the different tissues of the third instars of grain aphid, such as head, alimentary canal and fat body without alimentary canal.

To screen for potential RNAi target genes in grain aphid, 16 candidate genes which were highly expressed (based on their RPKM values) in both Chuli and Duizhao, or only expressed in one treatment, were selected for further confirmation of their actual expression levels in aphids after feeding on wheat plants and dsRNA artificial diet feeding assay (Table 2). The qRT-PCR analysis confirmed the DEGs analysis results of these selected genes in grain aphid upon feeding on wheat plants, with the relative expression levels of 8 unigenes (8273, 11975, 23028, 23235, 26956, 27882, 28447 and 28469) altered very significantly after feeding (**Student’s t-test, n = 3, p < 0.01), whereas that of another 4 unigenes (8279, 21088, 21789 and 29689) also changed significantly but had a lower probability value (*Student’s t-test, n = 3, p < 0.05) (Figure 3). Furthermore, semi-quantitative RT-PCR analyses of different aphid tissues (head, alimentary canal and fat body without alimentary canal) showed that the selected unigenes were expressed in the tested tissues at different levels except for unigenes 11975, 23235 and 27357 which could not be detected in head samples (Figure 4).

**Table 2:** Nr annotations and RPKM values of 16 selected candidate RNAi target genes in alimentary canal of grain aphid

**Unigene no.**	**Nr-annotation**	**RPKM value of the unigene in Chuli**	**RPKM value of the unigene in Duizhao**	**Log 2 ratio (Chuli RPKM/Duizhao RPKM)**
Unigene_6994	Synaptotagmin 4 [*Acyrthosiphon pisum*]	54.8647	35.322	0.64
Unigene_ 8273	Similar to cuticular protein CPG12 [*Acyrthosiphon pisum*]	1454.892	641.2085	1.18
Unigene_ 8279	Similar to CG10625 [*Acyrthosiphon pisum*]	1967.559	554.559	1.83
Unigene_11975	Hypothetical repeat protein [*Leishmania infantum*]	17.2449	4.6812	1.88
Unigene_21088	cytochrome c oxidase subunit VIIc precursor [*Acyrthosiphon pisum*]	3463.178	7555.03	−1.13
Unigene_21789	No orthologs identified	1987.456	886.7044	1.16
Unigene_23028	Similar to zinc finger protein [*Acyrthosiphon pisum*]	6.0578	0.0	Only in Chuli
Unigene_23042	Similar to AGAP007992-PA, partial sequence [*Acyrthosiphon pisum*]	6.0346	0.0	Only in Chuli
Unigene_23235	Similar to defective proboscis extension response [*Tribolium castaneum*]	5.4287	0.0	Only in Chuli
Unigene_ 26956	No orthologs identified	0.0	98.1775	Only in Duizhao
Unigene_ 27357	Similar to proteophosphoglycan ppg1 [*Acyrthosiphon pisum*]	1481.846	525.2179	1.49
Unigene_ 27882	Similar to neurofilament, heavy polypeptide isoform 2 [*Acyrthosiphon pisum*]	1602.747	751.6207	1.09
Unigene_ 28447	No orthologs identified	1861.09	494.6134	1.91
Unigene_28469	No orthologs identified	2775.923	174.3591	3.99
Unigene_ 28555	ACYPI002010 [*Acyrthosiphon pisum*]	2321.885	681.4342	1.76
Unigene_ 29698	No orthologs identified	0.0	98.4885	Only in Duizhao

### Screening for potential RNAi targets using an artificial feeding assay and the effects of dsRNAs on aphid development and mortality

**Figure 5:**
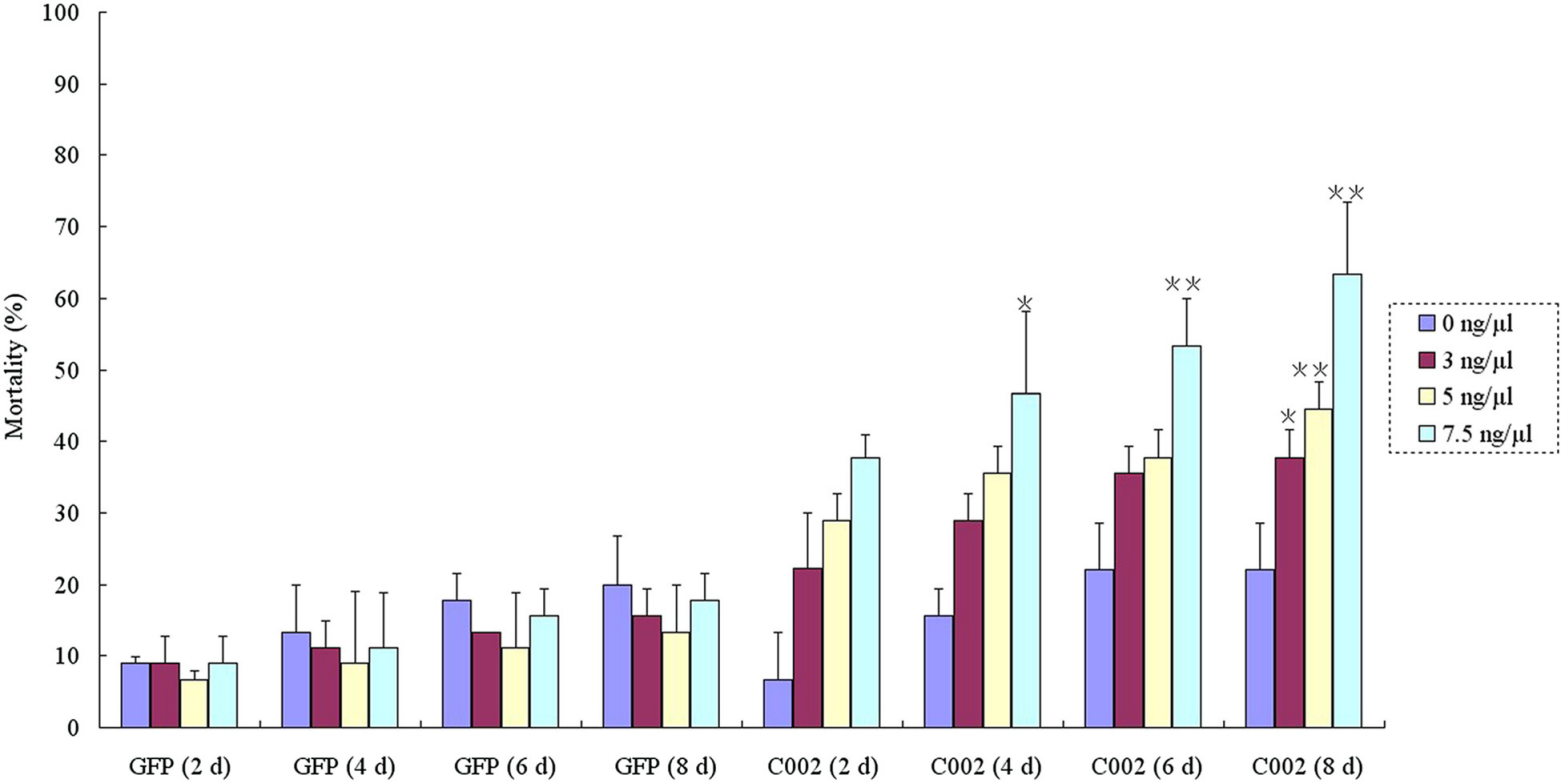
**The effects of different concentration of *C002 *dsRNA at different time point on the mortality of third instars of grain aphid.** The blank artificial diet without dsRNA was used as control. The aphid survival was monitored in an 8 d period. (*Student’s t-test, n = 3, p < 0.05; **Student’s t-test, n = 3, p < 0.01).

**Figure 6:**
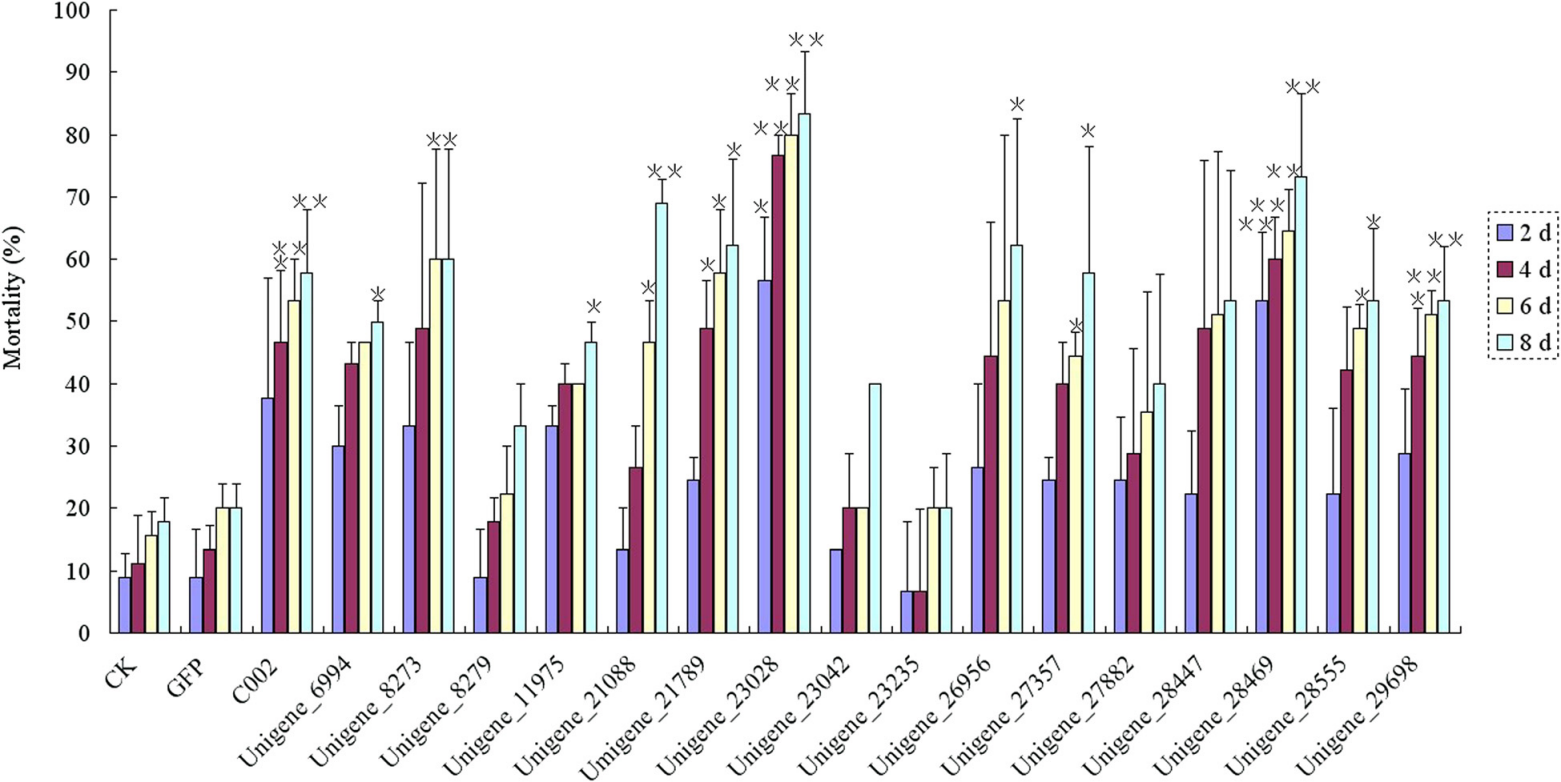
**The mortality of the third instars of grain aphid feed on artificial diet added with dsRNA of selected candidate RNAi target genes.** CK, represented the artificial diet control without dsRNA. The dsRNAs of sixteen candidate target genes along with that of *C002* gene were added to the artificial diet at a concentration of 7.5 ng/μl, respectively. The survival of the instars was monitored in an 8 d period. (*Student’s t-test, n = 3, p < 0.05; **Student’s t-test, n = 3, p < 0.01).

To screen for potential RNAi target genes in plant-mediated RNAi for aphid control in wheat, dsRNAs of the 16 candidate RNAi target genes listed above was synthesized in vitro and incorporated into an artificial diet. *C002* is a gland-specific gene first isolated from the pea aphid and knock-down of this gene led to the death of pea aphid and improved peach aphid resistance in transgenic tobacco plants [22,26]. A homologue of *C002* from grain aphid was identified and named as *SaC002* in this study (data not shown). Then, a 284 bp dsRNA was in vitro synthesized and used as a positive control to test the efficacy of other candidate RNAi target genes in this study. First, in order to test the efficacy of dsRNA on the mortality of aphid and its specificity, the effects of different concentration of *C002* dsRNA and *GFP* dsRNA added to the artificial diet on the mortality of third instars of grain aphid at different time points were investigated. As shown in Figure 5, compared with blank artificial diet without any dsRNA (control), the *GFP* dsRNA in the artificial diet had no effect on the mortality of aphids, whereas the mortality of aphids correlated with both increased concentration of *C002* dsRNA and feeding time, reaching a very significant level at 8 d for the concentration of 5 ng/μl, and 6 and/or 8 d for 7.5 ng/μl. Therefore, we used the 7.5 ng/μl concentration of dsRNA to investigate the mortality of the third instars grain aphids feeding on artificial diet added with dsRNA of 16 selected candidate RNAi target genes. As indicated in Figure 5 and Figure 6, the dsRNA of *GFP* control had no effect on the mortality of aphid, indicating the lethality mentioned above was caused by sequence-specific effect of the dsRNAs rather than by the physical or chemical characteristics of dsRNAs per se. When 7.5 ng/μl dsRNA was added to the artificial diet, 4 d later, the mortality levels of aphids fed with dsRNAs of *C002* and unigenes 21088, 21789, 23028, 28469 and 29698 were between 60% and 90% after correction. These data were statistically significant when compared with control treatments without dsRNA or with *GFP* dsRNA, and led to even higher mortality and developmental stunting than the positive control *C002* at day 8. The dsRNAs targeting the rest of the selected unigenes such as 6994, 8273, 11975, 26956, 27357 and 28555 also led to significant mortality either at day 6 or 8. However, no big differences on mortality were found in the treatments with dsRNA targeting unigenes 8279, 23042, 23235, 27882 and 28447. In addition, except for unigenes 21088 and 23028 which were predicted to encode orthologs of a cytochrome c oxidase subunit VIIc precursor and a zinc finger protein in pea aphid, respectively, KEGG pathway analysis and BLASTX against the nr and ApdidBase database (http://www.aphidbase.com) didn’t reveal any orthologs of 21789, 28469 and 29698 unigenes, suggesting they were novel or newly annotated genes/transcripts identified in grain aphid.

**Figure 7:**
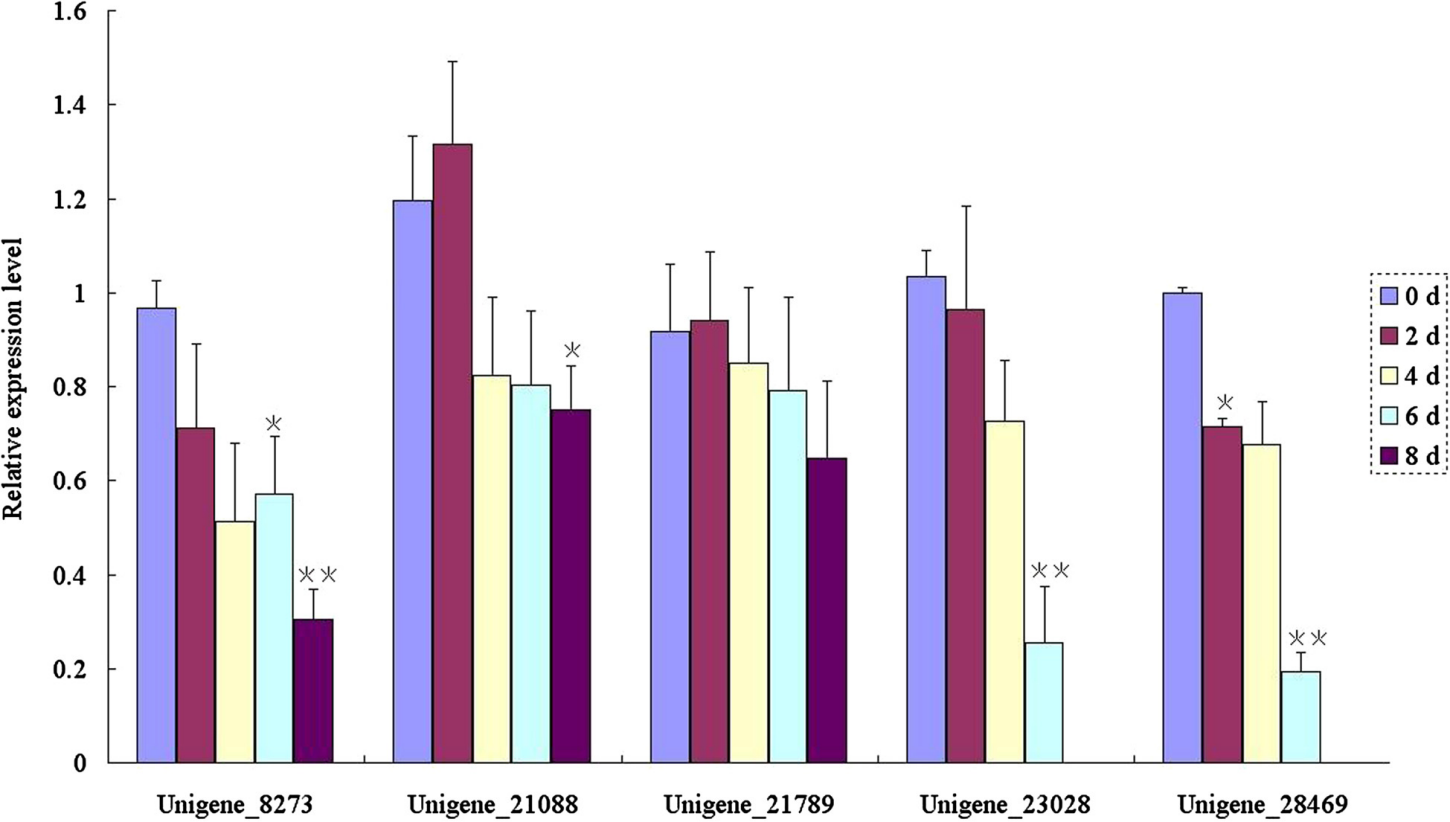
**The relative expression levels of 5 candidate RNAi target genes upon feeding on artificial diet containing dsRNAs at different time point.** The dsRNAs were supplied to the artificial diet at concentration of 7.5 ng/μl. qRT-PCRs were performed using the total RNA from the survived instars at different time point after feeding on artificial diet containing the respective dsRNAs. The relative expression levels of 5 RNAi target genes were monitored at indicated time point in an 8 d period. The expression of Unigene 8273, Unigene 23028 and Unigene 28469 was knocked down significantly after dsRNA feeding at day 6, with the latter two genes completely silenced at day 8. (*Student’s t-test, n=3, p<0.05; **Student’s t-test, n=3, p<0.01).

To investigate the correlation of larval mortality with the relative expression level of the respective target gene and to verify that administration of dsRNA does not affect the expression of household genes such as *actin*, five candidate RNAi target genes including the unigene 8273 which displayed highly up-regulated profile upon feeding on wheat plants (Figure 3) and other four unigenes 21088, 21789, 23028 and 28469 (Figure 6) were selected to detect their relative expression levels upon feeding on artificial diet added with the respective dsRNAs at different time point by using the aphid *actin* gene as internal control. The dsRNAs were supplied to the artificial diet at concentration of 7.5 ng/μl. qRT-PCRs were performed using the total RNA from the surviving instars at different time points after feeding on the artificial diet. The relative expression levels of 5 RNAi target genes were monitored at indicated time point in an 8 d period. As indicated in Figure 7, the expression of unigenes 8273, 23028 and 28469 was knocked down significantly after dsRNA feeding at day 6, with the latter two genes completely silenced at day 8 (**Student’s t-test, n = 3, p < 0.01). The expression levels of unigenes 21088 and 21789 increased 2 days after dsRNA feeding, and decreased in the following days, with that of unigene 21088 knocked down significantly at day 8 too (*Student’s t-test, n = 3, p < 0.05). These results indicated that the mortality and developmental stunting caused by feeding these dsRNAs was due to the down-regulation and even silencing of the respective target gene. Combined with the results of mortality analysis, we would like to propose that unigenes 8273, 21088, 23028, 28469 and 29698 would be good RNAi targets for grain aphid control through plant-mediated RNAi strategy in agricultural practice.

### Spread of fluorescence in the whole aphid body after feeding with labelled dsRNA

**Figure 8:**
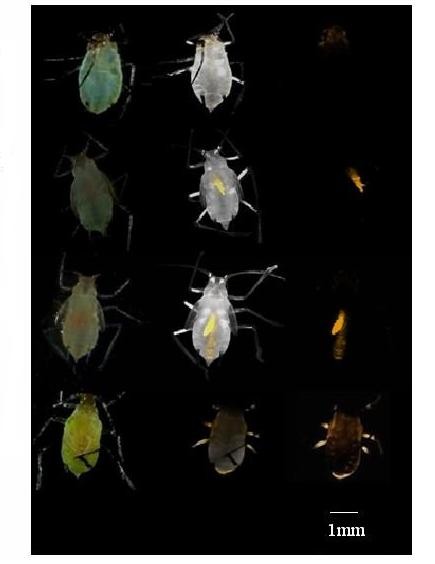
**A systemic RNAi effect in grain aphid observed by using the fluorescent labeled dsRNA added to the artificial diet.** The fluorescent labelled dsRNA were added to the artificial diet at a final concentration of 7.5 ng/μl. Fifteen third instar grain aphids were inoculated. **(A)** 1-2 h later, the fluorescence signal mainly observed in the mouthpart. **(B)** and **(C)** 3 h to 5 h later, strong fluorescent signals was mainly centralized in the midgut. **(D)** In the following time period of artificial diet feeding (6-24 h period), the fluorescent signal began spread around the whole body tissues. From A to D, the left panel of each line was observed under stereo microscope (SM), the middle panel of each line was on bright field (BF) and right panel was on 559 nm (Cy3) under Confocal Fluorescent Microscopy FV1000.

For the artificial feeding assay, the mid-gut is the primary target organ. Thus it would be interesting to know if the silencing signal would spread from midgut to other tissues in aphid, causing systemic RNAi. To investigate the systemic long-lasting RNAi effect, CTP labelled with Cy3 was added during the dsRNA synthesizing of unigene 23028. After removing the residual DNA and single strand RNA by DNase and RNase A digestion, the dsRNA was purified by MEGAclearTM columns (Ambion) and supplied to the artificial diet at a concentration of 7.5 ng/μl. The fluorescence signals were observed in a 24 h period. As indicated in Figure 8, 2 h later, the fluorescence signal was mainly observed in the mouthparts, indicating the start of dsRNA feeding (Figure 8A). Three to four hours later, strong fluorescent signals were observed, mainly in the midgut with some also observed in the intestine part of the digestion system (Figure 8B, 8C). In the time period of dsRNA artificial diet feeding experiment, namely 12, 18 or 24 h, the fluorescent signal spread around the whole body tissues (Figure 8D). This result showed the evidence for the first time that fluorescent labelled 23028 dsRNA could be taken up through the digestive system and was not localized to the midgut (the site of dsRNA delivery) and temporally limited. It demonstrates that dsRNA could penetrate the perimicrovillar membrane and spread to the whole body tissues in grain aphid, then lead to a down-regulation/knock out of the target gene expression and finally to the development retarding and/or death (Figures 6, 7).

## Discussion

Following with the release of the first pea aphid genome in 2010, the aphid community is gearing up for generating genome information for additional aphid species such as green peach aphid, green bug, Russian wheat aphid, potato and cotton aphid, and the pursuit of high-resolution comparative genomic and evolutionary analyses [37]. However, a comprehensive view of the molecular profile of grain aphid, a diverged species from pea aphid with host plants adaptation mainly to cereal crops has not been documented so far. The alimentary canals of aphids play a crucial role in ingestion and digestion. This study compared two transcriptomic profiles of the alimentary canals of grain aphid, either with, or without the influence of feeding on wheat plants. 5490 unigenes were found to be differentially expressed, with 2918 genes up-regulated and 2572 genes down-regulated (See Additional file 1). While there were no significantly enriched cellular components showing differential expression, the genes involved in three molecular functions were found to be enriched upon feeding such as NADH dehydrogenase activity, oxidoreductase activity, and genes involved in eight biological processes were significantly enriched upon feeding on wheat plants such as generation of precursor metabolites and energy, oxidation-reduction process, energy derivation by oxidation of organic compounds and etc. (See Additional file 2). Furthermore, among the 2021 unigenes involved in ‘Metabolic pathways’, 420 showed differential expression, which accounted for 18.09% of total DEGs (2322) with pathway annotation (See Additional file 3). These results suggested that upon feeding on wheat plants, these diversified genes and/or pathways played important roles in nutrition ingestion and digestion in aphid. The obtained transcriptome profile of the alimentary canal of grain aphid upon feeding on wheat plants facilitates our understanding of the molecular mechanisms underlying feeding, ingestion and digestion.

For species without sufficient genomic information, transcriptome profiling through RNA-seq could provide a mass-screening approach to identify candidate genes for RNAi targeting for potential application in pest insect control [32]. In this study, we chose the candidate RNAi targets based on comparison of the transcriptome profiling data of the alimentary canals of grain aphids pre- and post feeding on wheat plants. Among 5490 differentially expressed unigenes detected in grain aphid upon feeding, 16 candidate genes which were highly expressed (based on their RPKM values) in both Chuli and Duizhao, or only expressed in one treatment, were selected for the dsRNA artificial diet feeding assay (Table 2). Of these, 5 were ultimately identified as effective potential RNAi targets (Figure 6 and Figure 7). Successful and unsuccessful RNAi experiments have been also observed in a number of lepidopteran species [38]. In lepidoptera, out of 130 genes used for the dsRNA artificial diet feeding analysis, only 38% were silenced at high levels while 48% and 14% of the genes failed to be silenced or were silenced at low levels, respectively. The reason for the low rate of silencing may be due to the efficiency of RNAi-mediated knockdown appears to depend on the identity and nature of the target gene. The type of gene to be silenced can significantly affect the outcome of an RNAi experiment [38]. The susceptibility of different targets to RNAi effects also shows considerable variation in model species [9]. Some targets have proved to be completely refractory to suppression as observed in most of the neuronal expressed genes in *C. elegans*[36]. In this study, we observed the same phenomena and noticed that not all the dsRNAs of the candidate unigenes tested could lead to the knock-down of target genes and the developmental stunting or death of aphids. Among the selected 16 up- and down- regulated genes, only 5 of them were effective RNAi targets (Figure 6). As indicated in Table 2, except for unigenes 23028 and 29698 which only expressed in Chuli or Duizhao, respectively, the rest of RNAi target genes such as 21088, 21789 and 28469 were highly expressed in the alimentary canal in either Chuli or Duizhao or both treatments (Table 2). This suggested that the dsRNAs of highly expressed genes involved in ingestion and digestion might achieve more effective knock-down or silencing of the target genes and higher mortality of aphid.

Furthermore, for potential RNAi target selection in plant-mediated RNAi for aphid control, we need to keep in mind that the silencing must be highly specific for the intended target gene. The risk of unintended cross-species silencing would be a major biosafety concern in the future application of RNAi mediated aphid resistance. It is therefore obvious to select highly insect-specific genes with no good match to sequences in non-target organisms such as the donor plants for engineering, the natural enemies of the target pest or humans and animals that may consume the crop as food or feed. Of the five effective RNAi targets identified in this study, three unigenes 21789, 28469 and 29698 had no orthologs identified (Table 2), suggesting they were novel genes/transcripts identified in grain aphid for the first time, and could be potential RNAi target genes in managing aphid resistance in transgenic wheat plants. Therefore, massive screening and careful selection of the RNAi target genes would be essential for the future application of plant-mediated RNAi for aphid control.

In artificial feeding assays, the concentrations of dsRNAs may be a determing factor on the final RNAi effect, either on in vitro artificial feeding assay or in planta through plant-mediated RNAi for aphid control. The occurrence of RNAi effect either through feeding or injection depends on both the gene targeted and the insect species investigated. For example, a great variation exists among different lepidopteran species with respect to their sensitivity to systemic RNAi and variable levels of silencing can occur at very different concentrations of dsRNA. It is not true that exceeding the optimal concentration results in more silencing [25,38,39]. In a few species, including *H. cecropia*, *Antheraea pernyi* and *M. sexta*, high levels of silencing can be achieved by application of very low amounts of dsRNA (less than 10 ng per mg tissue in injection experiment) [40-42]. For the dsRNA feeding experiments, 15 insect species (representing 7 different orders), were investigated, and the amount of dsRNA applied varied from 5.4 ng/cm^2^ to 80 μg (no indication of the concentration) [12]. In pea aphid, lethal effects were achieved with different concentrations of dsRNA or SiRNA used for injection or feeding. For example, each aphid was injected with 5 nl *C002* SiRNA (10 μg/μl) led to knockdown of *C002* gene [22]. So far, dose–response relationships using lower concentration of dsRNA to establish the sensitivity to RNAi has not been reported in insects [38]. In this study, by using *SaC002* as a positive control, a clear dose–response relationship was established between the relative lower concentration of dsRNA and the aphid’s sensitivity to RNAi in artificial diet feeding bioassay in grain aphid (Figure 5). Based on this experiment, when dsRNAs were added at a concentration of 7.5 ng/μl, a lethal effect had been achieved for 5 potential RNAi target genes, with significant effects on mortality observed 4 d after feeding with dsRNAs targeting unigenes such as 23028, 29698 and 2 d for 28469, which performed even better than *C002* dsRNA (Figure 6). The lethal efficacy of the relative low concentration of dsRNA (7.5 ng/μl) observed in this study will not only maintain the minimal risk of non-specific effects but also facilitate the application of plant-mediated RNAi silencing of these target genes for aphid control in agricultural practice.

Systemic RNAi encompasses both cell-autonomous and environmental RNAi in which the silencing signal is transported from the cell in which the dsRNA is applied or expressed to other cells or tissues [12]. Small RNA pathways are highly conserved in animals including aphids [12,43]. However, although orthologs of RNA-dependent RNA polymerase (RdRP) are present in nematodes, plants and higher animals, the presence of RdRP was never confirmed in insects [20,28,44]. Therefore, the absence of dsRNA amplification and RdRP in insects suggests that gene knockdown effects exhibited by injecting and/or feeding dsRNA to insects would be temporary, limited to cells that have taken up dsRNA and would require continuous input of dsRNA to persist, or in other words, systemic RNAi probably does not exist in insects [9,45]. Nevertheless, systemic RNAi has been demonstrated in some insect species, such as *Hyalophora cecropia* and *B. mori*, in which injection of dsRNA into the pupa can result in phenotypic effects in developing embryos, indicating dsRNA uptake by the developing oocytes of the pupa, and knockdown of a gene expressed in adult antenna of light brown apple moth (*Epiphyas postvittana*) could be achieved through feeding dsRNA to larvae, demonstrating a persistence of the RNAi signal throughout the larval and adult stages and systemic spread of RNAi signal from the gut to the antennae [38,46]. Successful knockdown of target genes through RNAi was also observed in pea and peach aphid [22-25], however, no direct evidence for the existence of systemic RNAi has ever been presented in aphid species. Given that *MpC002* expression, which occurs predominantly in the salivary glands, is knocked down by up to 60% in peach aphid upon feeding on dsRNA transgenic tobacco plants, the silencing signal appears to spread between organs in aphid species [26]. In this study, CTP labelled with Cy3 was added during the synthesis of dsRNA designed to unigene 23028 and the long-lasting RNAi effect was observed. The fluorescence signal were observed first in the mouthparts, and then centralized in the midgut and finally it spread through the whole body (Figure 8). BLAST analysis of the transcriptome data obtained in this study against the public databases revealed the presence of some RNAi core machinery elements such as Argonaute-2B, Dicer-1, SID-1 and also TAR RNA binding protein (TRBP), which function in assisting the RISC formation (data not shown). However, the mechanisms underpinning the spread of fluorescence signal still need to be further investigated, for example, the spread of fluorescence signal is through the aphid’s circulatory system or the in vivo amplification of siRNA, in which cells or tissues the target genes were silenced, and whether the proposed receptor mediated endocytosis or the transmembrane channel-mediated uptake are the mechanism leading to the persistence of RNAi effect.

Nevertheless, we here identified 5 novel and effective RNAi targets in grain aphid based on comparison of the transcriptome profiles of the alimentary canal of grain aphid upon feeding on wheat plant and presented the first evidence that fluorescent labelled dsRNA could be taken up through the digestive system and was not localized to the midgut (the site of dsRNA delivery) and temporally limited, could spread to the whole body tissues in grain aphid, then lead to a down-regulation/knock out of the target gene expression and finally to the development retarding and/or death of grain aphid. This laid a fundamental basis for future plant-mediated RNAi for aphid control in agriculture.

## Conclusions

In this study, we performed transcriptome assembly and gene expression analysis of the alimentary canals of pre- and post-feeding grain aphids on wheat plants using Illumina’s RNA sequencing. We obtained 30,427 unigenes which were expressed in grain aphid alimentary canal. Furthermore, of 5490 unigenes differentially expressed upon feeding, diverse genes and/or pathways involved were identified. Moreover, among the selected 16 up- or down-regulated unigenes for dsRNA artificial diet feeding test, the dsRNAs of 5 unigenes led to higher mortality and developmental stunting due to the down-regulation of the target gene expression. Finally, by adding the fluorescent labelled dsRNA into the artificial diet, the spread of fluorescent signal in the whole body was observed. The obtained transcriptome profile of the alimentary canal of grain aphid upon feeding on wheat plants would facilitate our understanding the molecular mechanism underlying feeding, ingestion and digestion. Furthermore, the identified 5 novel and effective RNAi target genes would provide a fundamental basis for aphid control in wheat through plant mediated RNAi strategy.

## Methods

### Plant and insect materials

Wheat: 15–20 seeds of wheat cv. Kenong 199 (a low susceptible wheat variety to aphid infestation) were planted in pots (10 cm diameter) that were kept in culture room at 22°C, 40%–60% relative humidity and a photoperiod of 16:8 (L:D). Plants at the two-leaf stage in each pot enclosed in Perspex tubes which were sealed with porous plastic sheeting were used for the aphid bioassay.

Aphids: Apterous adult grain aphids (*S. avenae*) derived from a single clonal lineage reared on wheat plants was placed in cages for 24 h to produce nymphs. Ten neonate nymphs produced in the 24 h period were transferred into fresh wheat plants in each pot. 12 days later, the third intars of these aphids were selected and subjected to artificial diet feeding experiment.

### Wheat plant feeding assay and dissection of aphid alimentary canal upon feeding

The aphids derived from a single clonal lineage were used for the following analyses. Four hundred grain aphids at different development stages, for example, 80 first instars, 80 second instars, 80 third instars, 80 fourth instars and 80 adults were selected and kept in a glass vial for in vitro bioassay without any artificial diet for a period of 6 h. Then, half of them was moved to fresh wheat plants and subjected to feeding. Six hours later, the aphid alimentary canal from pre- and post-feeding aphid treatments were dissected from the whole body on ice under the microscope. The samples were immediately frozen in liquid nitrogen, and stored at −80°C before RNA extraction.

### Sample collection and RNA isolation

Total RNA was isolated using a Qiagen RNA Extraction kit according to the manufacturer’s instructions (New England BioLabs). It was treated with RNase-free DNase I for 30 min at 37°C to remove residual DNA, and mRNA was isolated from DNA-free total RNA using Dynabeads mRNA Purification Kit (Invitrogen).

### cDNA synthesis, sequencing and data analysis of RNA-seq

Beads with Oligo(dT) were used to isolate poly(A) mRNA after total RNA collected from grain aphid guts pre- and post feeding on wheat plants. Fragmentation buffer was added for interrupting mRNA to short fragments. Taking these short fragments as templates, random hexamer-primer was used to synthesize the first-strand cDNA. The second-strand cDNA was synthesized using buffer, dNTPs, RNaseH and DNA polymerase I, respectively. Short fragments were purified with QiaQuick PCR extraction kit and resolved with elution buffer for end reparation and the natural addition of the poly(A). Then the short fragments were joined with sequencing adapters. After the agarose gel electrophoresis, the suitable fragments were selected as templates for the PCR amplification. At last, the library was sequenced using Illumina HiSeq™ 2000. Short read sequences were de novo assembled using SOAP de novo program [47]. Short clean reads were loaded into computer and the unambiguous sequence fragments were obtained as contigs. Then the reads were realigned onto the contigs according to the paired-end information and the unique contigs were joined into scaffolds. Finally, the intra-scaffold gaps were filled using the paired-end extracted reads. Those sequences which can not extend to any direction were unigenes. The unigenes were analysed using BLASTX against the database of nr, Swiss-Prot, COG, GO and KEGG for functional annotations. The expression levels of unigenes were estimated by RPKM method [48].

### dsRNA synthesis

dsRNAs were synthesized using the MEGAscript RNAi kit (Ambion, Huntingdon, UK) according to manufacturer’s instructions. T_7_ promoter sequences were tailed to each end of DNA template by PCR amplifications. Double stranded GFP (dsGFP) was generated using pPigbacA3EYFP as template and was used as a negative control in the experiments. All the primer sequences were listed in Table 3. Template DNA and single-stranded RNA were removed from the transcription reaction by DNase I and RNaseA treatment. dsRNA was purified using MEGAclearTM columns (Ambion) and eluted in 100 μl nuclease free water. dsRNA concentrations were measured using Biophotometer (Eppendorf, Germany).

**Table 3:** Primer sets used for qRT-PCR analysis of the selected candidate genes upon aphid feeding on wheat plants and in vitro dsRNA synthesis of 16 candidates

**Unigene no.**	**Forward primer**	**Reverse primer**
Unigene_6994	TAATACGACTCACTATAGGGAGCTGAAAAGTGAAATGATGGTGGTC	TAATACGACTCACTATAGGGTTGCCAAACTGGAAAATCG
Unigene_ 8273	TAATACGACTCACTATAGGGAGTGCGTCAGATAGTCCGGCACC	TAATACGACTCACTATAGGGCACCATTCAAATCCGTTTCTTACAGC
Unigene_ 8279	TAATACGACTCACTATAGGGAGAAAGCAATGCTGTGGCAGAAAC	TAATACGACTCACTATAGGGGTGGTCGTTCTTTCAGTGATGG
Unigene_11975	TAATACGACTCACTATAGGGAGCGCTGTTGGTGCTGGTGGTGATG	TAATACGACTCACTATAGGGTCGTTGGACGGTTTGGTCGATGTCT
Unigene_21088	TAATACGACTCACTATAGGGAGGTTTAACGAATCGGACCATCAGGAA	TAATACGACTCACTATAGGGAAAATATGATCGCCTCAAGGGGACT
Unigene_21789	TAATACGACTCACTATAGGGAGTACCGTCCGCAAATGGTTGG	TAATACGACTCACTATAGGGTCCGAAGTCACATGTTCGCA
Unigene_23028	TAATACGACTCACTATAGGGAGGTTATTGTTGAACCCTTCTGACACG	TAATACGACTCACTATAGGGGAAGGATTAGACGATATTTTGGTG
Unigene_23042	TAATACGACTCACTATAGGGAGGTTCCATGACAAGCCGGATAC	TAATACGACTCACTATAGGGGACTCAAGTGCCTGGTGGGT
Unigene_23235	TAATACGACTCACTATAGGGAGGGTGCCCGACTCGGTTTTCTCC	TAATACGACTCACTATAGGGGCTGCCCAAGCCAACATCCAC
Unigene_26956	TAATACGACTCACTATAGGGAGCCGTGATTCTCCTGCGTCTGCT	TAATACGACTCACTATAGGGAGTCTTCGCCACCGCCGTTT
Unigene_27357	TAATACGACTCACTATAGGGAGATGGTTTTATGGTTGGCTTTAGTCCAG	TAATACGACTCACTATAGGGCCGCGTTCGCATTTCGTTTT
Unigene_27882	TAATACGACTCACTATAGGGAGAGGTATCACCCGCCGTACC	TAATACGACTCACTATAGGGGAACAGCTGGTTCTGCGAAA
Unigene_28447	TAATACGACTCACTATAGGGAGTGGCCCGTACGACACACCGA	TAATACGACTCACTATAGGGCGGACGATGTGCCGGGTGAC
Unigene_28469	TAATACGACTCACTATAGGGAGCAAATGAAACGCCGTATTTGATAA	TAATACGACTCACTATAGGGCGTGCCTACCGTATTCGACAAT
Unigene_28555	TAATACGACTCACTATAGGGAGGGGTTAGACTTGCCCGAAACTGATG	TAATACGACTCACTATAGGGTCCATGTTGGCAGTCCAACCGT
Unigene_29698	TAATACGACTCACTATAGGGAGCGTCGCCTTGGTGAGCCTTTAC	TAATACGACTCACTATAGGGCGGCGGACGGGTGAGTAATG
*Actin*	CCGAAAAGCTGTCATAATGAAGACC	GGTGAAACCTTGTCTACTGTTACATCTTG
*GFP*	TAATACGACTCACTATAGGGAGGCAGTGCTTCAGCCGCTACCC	TAATACGACTCACTATAGGGCCTTGATGCCGTTCTTCTGCTTGTC

Note: T_7_ promoter sequences were tailed to each 5’ end of primers and underlined. The sequences without T_7_ promoter sequence tails were used for qRT-PCR analysis of the selected candidate genes upon aphid feeding on wheat plants.

### Fluorescent labelled dsRNA to observe the RNAi effect

The dsRNA synthesis procedure was the same as described above except that 0.25 ml 10 mM fluorescent dCTP labelled with Cy3 was added, taking advantage of character that T_7_ RNA polymerase could take dNTP as substrates for synthesis RNA [49]. Then the labeled dsRNA was purified by using MEGAclearTM columns (Ambion) and used as supplement to an artificial diet at a concentration of 7.5 ng/μl. The aphids fed with Cy3 labelled dsRNA were observed under Confocal Fluorescent Microscopy FV1000 (Olympus) every 2 h in a 24 h period.

### Insect bioassay

The artificial diet bioassay was performed according to the following procedure. Liquid artificial diet was prepared as described by Whyard et al. (2009) [23] and filtered through 2 μm membrane, and dispensed in 200 μl aliquots and stored at −20°C before the artificial diet feeding assay. Glass vials (2 cm in diameter) for aphid artificial double-membrane feeding assay were sterilized and one opening completely sealed with parafilm before sample application. 50 μl of test samples containing either nuclease-free water or dsRNA were added to the 1 ml artificial diet to the final concentration of 0, 3, 5 and 7.5 ng/μl, respectively. 200 μl aliquots of samples were pipetted onto the parafilm and another layer stretched on top to form a sachet and made sure the liquid diet distributed evenly. Fifteen third instar aphids were added per vial with a fine paintbrush. Then, another opening was covered with a piece of sterilized gauze. At least 3 replicates were set up for each experimental group. The bioassay vials were put with the opening with gauze upside and incubated at 22°C-24°C, 40%–60% relative humidity and a photoperiod of 16:8 (L:D). For each treatment, the number of survival aphids was counted every 2 d in an 8 d period. The data were analyzed using one-way ANOVA to investigate the effects of the dsRNA treatment on mortality of aphids compared to the untreated control (*Student’s t-test, n = 3, p < 0.05; **Student’s t-test, n = 3, p < 0.01).

### Quantitative real-time PCR (qRT-PCR)

Expression levels of the 16 up- or down- regulated selected unigenes in grain aphid upon feeding on wheat plants according to the RPKM were re-evaluated by qRT-PCR. The sequences of primer sets were provided in Table 3 except for the T_7_ promoter sequences were not added. A qRT-PCR assay for multiple genes was performed with the SYBRH Premix Ex Taq TM II (TaKaRa). Grain aphid *actin* expression levels were used to normalize *Ct* values obtained for each gene. qRT-PCR was carried out using a Mastercycler ep realplex instrument (Eppendorf). Data analysis methods were described as the reference [50]. To assess the extent of RNAi, RNA was extracted from pools of 10 dsRNA-treated and survived aphids using a Qiagen RNA Extraction kit. Primer sets were synthesized as listed in Table 3 with no T_7_ promoter sequences tailed. The samples were then treated with DNAase I (Invitrogen) to remove any genomic DNA contamination, and were used with Superscript II reverse transcriptase (Invitrogen) to make first strand cDNA using random primers. qRT-PCR reactions and statistical analysis were the same with mentioned above except for grain aphid *actin* expression level was used to normalize *Ct* values obtained for each unigene.

## Abbreviations

BLAST: Basic local alignment search tool; COG: Clusters of orthologous groups; DEGs: Differentially expressed genes; dsRNA: Double-stranded RNA; ESTs: Expressed sequence tags; GO: Gene ontology; KEGG: Kyoto encyclopedia of genes and genomes; Nr: Non-redundant protein database; Nt: Nucleic acid database; qPCR: Quantitative polymerase chain reaction; RNAi: RNA interference; RPKM: Read per kb per million; RT-PCR: Reverse-transcript polymerase chain reaction.

## Competing interests

The authors declare that no competing interests exist. The funding organizations had no role in study design, data collection and analysis, decision to publish, or preparation of the manuscript.

## Authors’ contributions

Conceived and designed the experiments: LQX, YZM and HDJ. The experiments were performed by MZ, HW and DHW. YWZ and QG analyzed the data. The manuscript was written by LQX, YZM and HDJ. All authors read and approved the final version of the manuscript.

## Supplementary Material

Additional file 1 **Summary of 5490 DEGs and their annotations.** The relative expression levels of DEGs and their annotations in the nr, COG, GO, and KEGG public databases were summarized. Among the 5490 differentially expressed genes, only 3805 were annotated in one of the above public databases. Click here for file

Additional file 2 **GO terms enriched in the differentially expressed genes.** The results were summarized in three main categories: biological process, cellular component and molecular function. GO analysis showed that three molecular functions were found to be enriched at p < 0.05 level, and eight biological processes were significantly enriched at p < 0.05 level in the alimentary canals of grain aphid upon feeding on wheat plants. Click here for file

Additional file 3 **KEGG pathways enriched in the differentially expressed genes.** KEGG pathway analysis showed that 420 unigenes which are involved in ‘Metabolic pathways’ showed differentially expressed manner, accounting for 18.09% of total DEGs (2322) with pathway annotation. Click here for file
